# Assessment of the Impact of Rapid Point-of-Care CD4 Testing in Primary Healthcare Clinic Settings: A Survey Study of Client and Provider Perspectives

**DOI:** 10.3390/diagnostics10020081

**Published:** 2020-02-01

**Authors:** Shabashini Reddy, Andrew Gibbs, Elizabeth Spooner, Noluthando Ngomane, Tarylee Reddy, Nozipho |Luthuli, Gita Ramjee, Anna Coutsoudis, Photini Kiepiela

**Affiliations:** 1 South African Medical Research Council, Durban 4000, South Africa; Shabashini.reddy@mrc.ac.za; 2 Wits Health Consortium, Parktown, Johannesburg 2091, South Africa; 3 South African Medical Research Council, Gender and Health Research Unit, Durban Centre for Rural Health, University of KwaZulu Natal, Durban 4000, South Africa; Andrew.gibbs@mrc.ac.za; 4 South African Medical Research Council, HIV Prevention Research Unit, Durban 3600, South Africa; Elizabeth.spooner@mrc.ac.za (E.S.); Gita.ramjee@mrc.ac.za (G.R.); 5 Occupational Health, Hillcrest, Durban 3610, South Africa; thando.frd.ngomane@gmail.com; 6 South African Medical Research Council, Biostatistics Unit, Durban 4000, South Africa; Tarylee.reddy@mrc.ac.za; 7 eThekwini Health Unit, Durban 4000, South Africa; Nozipho.Luthuli@durban.gov.za; 8 School of Clinical Medicine, University of KwaZulu Natal, Durban 4000, South Africa; coutsoud@ukzn.ac.za

**Keywords:** point-of-care CD4+ t testing, qualitative survey, acceptability, patients, healthcare providers, primary healthcare clinics

## Abstract

Background: The high burden of disease in South Africa presents challenges to public health services. Point-of-care (POC) technologies have the potential to address these gaps and improve healthcare systems. This study ascertained the acceptability and impact of POC CD4 testing on patients’ health and clinical management. Methods: We conducted a qualitative survey study with patients (*n* = 642) and healthcare providers (*n* = 13) at the Lancers Road (experienced POC) and Chesterville (non-experienced POC) primary healthcare (PHC) clinics from September 2015 to June 2016. Results: Patients (99%) at Lancers and Chesterville PHCs were positive about POC CD4 testing, identifying benefits: No loss/delay of test results (6.4%), cost/time saving (19.5%), and no anxiety (5.1%), and 58.2% were ready to initiate treatment. Significantly more patients at Chesterville than Lancers Road PHC felt POC would provide rapid clinical decision making (64.7% vs. 48.1%; *p* < 0.0001) and better clinic accessibility (40.4% vs. 24.7%; *p* < 0.0001) respectively. Healthcare providers thought same-day CD4 results would impact: Clinical management (46.2%), patient readiness (46.2%), and adherence (23.0%), and would reduce follow-up visits (7.7%), while 38.5% were concerned that further tests and training (15.4%) were required before antiretroviral therapy (ART) initiation. Conclusion: The high acceptability of POC CD4 testing and the immediate health, structural, and clinical management benefits necessitates POC implementation studies.

## 1. Introduction

South Africa has an estimated population of 58.78 million with 7.97 million living with Human Immunodeficiency Virus (HIV), of whom 20% are women of reproductive age (15–49 years) [1], while the highest HIV prevalence ((27%) is in the province of KwaZulu Natal [1]. In September 2016, South Africa [2] adopted the World Health Organization (WHO) recommendations of universal treatment to all adults living with HIV, regardless of CD4 count [3], resulting in more than 4.5 million people taking antiretroviral therapy (ART), making it the largest ART programme globally [4].

Conventional HIV treatment and care services provided at primary healthcare (PHC) clinics in the public service in South Africa are largely unable to cope with the volume of patients entering the system, resulting in delayed and missed opportunities for treatment and ultimately unacceptably high levels of morbidity and mortality [5].

Diagnostic CD4 testing performed by conventional flow cytometry is centralized and offsite, provided by the National Health Laboratory Service (NHLS), serving >80% of the population [6]. There are several drawbacks of conventional testing, both from the patient and laboratory perspective, viz:

- Risk of losing patients who may not return due to cost and distance; delays in diagnosis and treatment initiation;
- Patients who seek healthcare elsewhere become nontraceable, giving wrong addresses to clinics further away for eligibility resulting in unnecessary repeat testing and higher workloads in some PHC clinics [7];
- Incomplete or incorrect completion of request forms or labelling of test tubes;
- The rejection of sample quality (insufficient or clotted specimen);
- Specimen damage or loss through transport;
- Misplacing of printed laboratory results at the clinic [7,8].

Instituting point-of-care (POC) CD4 testing in PHC clinics with the availability of same day results has the potential to address a number of these challenges for both patients and the health system. Additionally, POC testing brings with it greater patient satisfaction and helps with the morale of healthcare providers doing away with the “frustration” associated with conventional testing [9]. Daneau et al. (2016) [10] stated that the only objection to finger stick POC CD4 testing was due to pain/soreness. Additionally, some patients may decline POC testing as they may not feel emotionally/psychologically ready to receive same-day results [11].

Implementation of POC studies has demonstrated the reduction of pretreatment loss to follow-up in Mozambique [12,13], acceleration of ART initiation, but not retention, in care at 12 months [14], and reducing the time to diagnosis of multidrug resistance tuberculosis (TB) in South Africa [15]. In simulated cohort models of HIV-infected adults and pregnant women, the provision of same-day CD4 results was shown to result in better clinical outcomes and cost savings over the long term (five years) [16,17]. Another study focusing on POC processes across multiple diseases found that other challenges and delays were created with respect to the continual interaction of patient and healthcare [7].

Barriers to POC implementation have also been documented [7,18] where it was shown that POC testing needs to be integrated efficiently into the clinical care pathways. Otherwise it can result in increasing waiting time [19] and length of clinic visit [7,19,20,21].

Several studies have assessed the acceptability of POC assays, such as CD4 testing [10] and the POC viral load (VL) early infant diagnosis (EID) [9] in patients, resulting in better clinical outcomes [22,23]. The weaknesses of prior qualitative research were the small sample size and self-selecting sample in a study [24] or a project recruiting a specific population within the PHC or a hospital [9,10]. The strength of our work offers an unbiased alternative perspective of the general patient population within the PHC who was willing to give consent when referred for phlebotomy. The other advantage is the comparison of two PHC clinics with differing POC testing experiences.

At the time of undertaking this qualitative survey study, several POC technologies (Alere PIMA^TM^ CD4, [25] TB LAMP [26], and EID [9]), were being evaluated at the Lancers Road PHC clinic. It was therefore an opportune time to explore the provision of same-day POC CD4 test result as patients could relate these results to their health. Although guidelines have changed and CD4 tests have been replaced with VL testing for treatment adherence, the data presented here remain relevant in understanding how nurses and patients interpret and make sense of POC in PHC settings. We therefore sought to assess the acceptability, understanding, and perceptions in both client and healthcare provider perspective on the usefulness and impact of rapid POC CD4 testing in a POC research “experienced” site (Lancers Road PHC) compared to a research “naïve” site (Chesterville PHC).

## 2. Materials and Methods

### 2.1. Study Design

This was a qualitative survey study determining the acceptability, understanding, and perceptions from the client and healthcare provider perspective of the impact of the provision of POC CD4 testing in a PHC clinic setting. A qualitative survey utilises open-ended questions (rather than closed yes/no or agree/disagree questions) and delivers this to a larger sample of participants than is typical in a qualitative study [27]. This allows for the assessment and quantification of a variety of opinions without providing a fixed set of opinions for responses.

### 2.2. Study Population

The study population consisted of a convenience sample of clients presenting at the Lancers Road and Chesterville PHC clinics under the eThekwini Health Unit from 25 September 2015 to 30 June 2016. Individuals (>18 years old) who were referred to the “blood room” for phlebotomy (both HIV-1 negative and HIV-1 positive) and were willing to provide informed consent were included in the study.

### 2.3. Study Setting

Lancers Road PHC clinic is a busy primary health clinic (PHC) facility under the eThekwini Health Unit, situated in the centre of the convergence of the taxi rank from all the outlying areas into the city of Durban. Chesterville PHC is situated within the Chesterville community, serving a population of 15,840 [28] and situated 13.0 km from the centre of Durban.

Lancers Road PHC was considered a POC research “experienced” site as different studies were being undertaken evaluating several POC tests including POC CD4, whereas Chesterville PHC was a research “naïve” site as far as POC testing was concerned.

### 2.4. PHC Clinic Procedures

Both PHCs offered all PHC services seeing 250–400 patients per day. This included HIV Counselling and Testing (HCT) for walk-in patients, with both clinics performing on average 600–700 HCT/month. Both PHC clinics provided basic education sessions every morning in the waiting room covering different topics:

Chronic care (diabetes; hypertension; cardiovascular diseases);
Antenatal (breastfeeding; pregnancy; immunisations for children);
Cancer (breast and ovarian);
HIV education.

**Figure 1:**
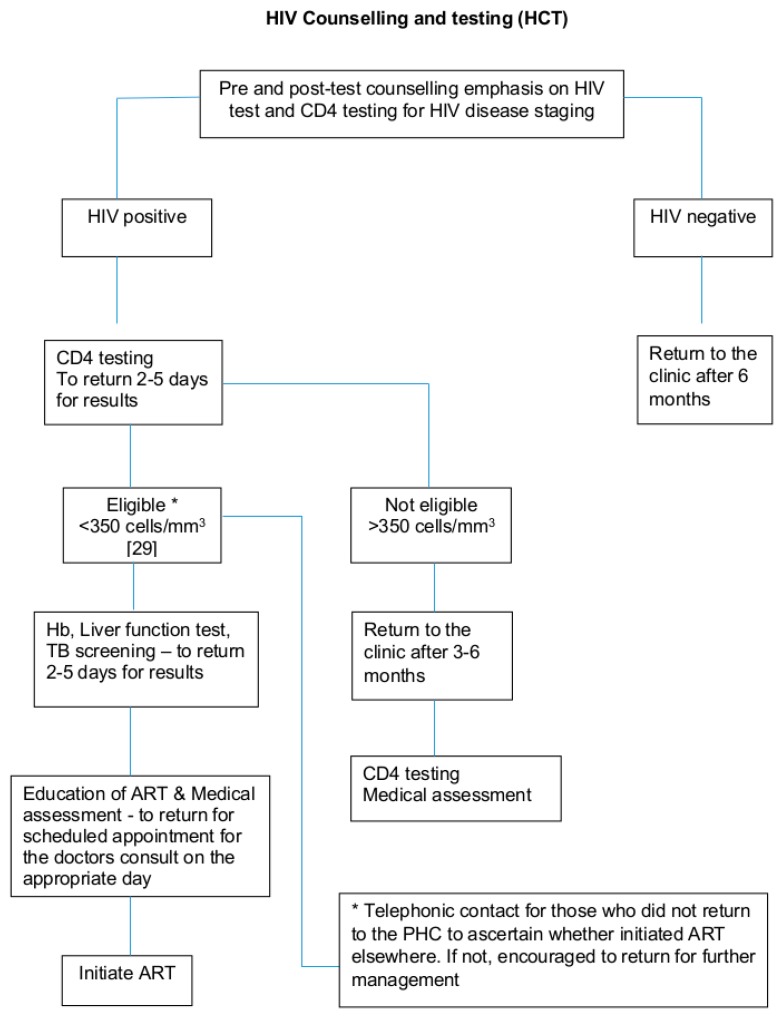
Schematic flow of the PHC clinic procedures with respect to HIV Counselling and Testing (HCT) [29].

The PHC clinic procedures with respect to HIV Counselling and Testing (HCT) are depicted in Figure 1.

### 2.5. Study Procedures

Patients’ requiring phlebotomy (both HIV-1 positive and HIV-1 negative) who were seen in the “blood room” by the counsellor/phlebotomist were approached by a study research assistant to participate in the study. Hence, there was no stigmatization for those that were HIV-1 infected as only the counsellor/phlebotomist and the patient knew what blood draw/s were required. They provided written informed consent, and then completed face-to-face questionnaires. If during enrollment patients asked what a POC test was, they were told, “It is a test you get back on the same day”.

Patient questionnaires focused on their understanding and perceptions of a POC laboratory on-site providing same-day results, their interpretation of a CD4 test, and whether they were ready to start ART if eligible. For each question (5 in total), we initially asked participants a yes/no closed question. We specifically asked five questions:

1. Are you happy to receive a CD4 test result on the same day? (*n* = 642)
2. Would you rather wait for a CD4 test result or return to the clinic another day?
3. How long did it take to get your CD4 test result?
4. Do you know what a CD4 test result means?
5. Are you ready to start ART if eligible?

After each closed item, we asked a single open-ended question, probing their answer. Participants had three lines to answer on, however most just recorded short answers.

For healthcare providers, we approached all within the PHC and requested their participation. Questionnaires were also administered to the healthcare providers. Healthcare provider questionnaires mainly focused on their perception of the usefulness of same-day CD4 results to patients and their interpretation of the meaning of a CD4 test result, as well as the impact that same-day CD4 results would have on their workload, patient clinical management, and administration of ART initiation. Similarly, for each question (5 in total), we asked healthcare providers initially a yes/no closed question. We specifically asked 5 questions:

1. Do you think it is beneficial for the patient to get their CD4 result on the same day?
2. What was the impact on your workload in giving CD4 results to the patient on the same day?
3. Does having a CD4 result on the same day help you with patient management?
4. Do you know what a CD4 test result means?
5. Were you able to administer antiretrovirals (ARVs) to the patient on the same day you had the CD4 test result?

After each closed item, we asked a single open-ended question probing their answer. Nurses had 5 lines to answer on.

### 2.6. Data Handling and Recordkeeping

Study records were maintained safely in a locked cabinet on-site for the entire study period. The risks of participation were minimal. Confidentiality was maintained by assigning each patient/healthcare provider a unique study number and using the study number as the sole patient/healthcare provider identifier. Patient responses were entered into a specific database, which was secured using password-protected access systems.

### 2.7. Ethics

The study was approved by the Medical Research Council Research Ethics Committee (EC017-6/2015) as well as the eThekwini Research Ethics Committee (No. M.1/1/2 2 September 2015).

### 2.8. Statistical Considerations

#### 2.8.1. Sample Size

The study was powered on patient acceptance of POC testing, which was assessed from the response to question 3.1 (Do you think it is a good idea to have a point of care laboratory in the clinic?). To detect a 90% POC test acceptance rate within a 7% margin of error at an alpha of 5%, 71 consenting patients were required. Assuming that 10% of HIV positive patients refused to consent to the study, approximately 80 HIV positive patients were required to reach the required sample size. Under the assumption that 25% of patients who were present for HIV testing were HIV positive, our sample size target was 320 patients in total at each PHC clinic, to be screened for entry into the study.

#### 2.8.2. Statistical Analysis

The analysis of categorical outcomes is presented as frequencies and percentages.

As this was a qualitative survey study, data from the questionnaires from patients from each PHC clinic and healthcare provider (all nursing staff at the Lancers Road and Chesterville PHC clinics) were computed using coding. The binomial test with normal approximation was used to test whether proportions observed differed significantly between clinics. A 5% level of significance was used. Data were analyzed using Stata version 13.

To understand the variation qualitatively in people’s responses, we did an open coding on participants’ written answers to each question. We then organized the different small codes into larger themes, which connected codes together to understand the responses of participants. Once we had done this, we allocated each participant a response code and calculated the percentage and number who provided each reason for their answer.

## 3. Results

There were 642 patients interviewed, 322 at Lancers Road PHC and 320 at Chesterville PHC, of whom 272/322 (85%) and 233/320 (72.8%) were women with a median age of 32 and 34 years at Lancers Road and Chesterville PHC, respectively. No one refused to participate.

The overwhelming majority (99.5%) of patients in both the research “experienced” (Lancers Road; 322/322 (100%)) and “naïve” (Chesterville; 318/320 (99.4%)) PHC clinics welcomed the receipt of same-day CD4 test results ((a) in Table 1). Qualitatively, there were three reasons why patients were happy to receive their results immediately: Three-quarters (73.8%) said it was so they could receive medical care and help immediately, including starting ARVs if necessary. A fifth (19.5%) reported that it would save them time and money, as they would not need to return to the clinic. Meanwhile, 6.4% reported that it would mean there would be no delay or loss of CD4 test results. Only 0.30% (*n* = 2) participants reported that they would not want this, because they needed time to consider the results.

Similarly, (b) in Table 1 presents participants’ responses to the question about whether they would rather wait for their CD4 results or return another day. As with the first question ((a) in Table 1), the vast majority (96.4%) would rather wait for their CD4 test result, rather than return another day. There was some variation in reasons between the two clinics. Just over half (56.4%) of the sample reported wanting to get their results quickly and being able to start treatment; however, significantly less (48.1%) at Lancers than at Chesterville (64.7%) (*p* < 0.0001) reported this. Clinic accessibility was also a challenge, but this varied by clinic with significantly more patients at the Lancers Road (40.4%) than Chesterville PHC (24.7%) having difficulty with clinic accessibility (*p* < 0.0001). One in twenty (5.9%) at Lancers clinic reported they would not have the anxiety of waiting for their results if they received them within the day. An overall minority (3.4%) at both PHC clinics preferred CD4 test results on a different day.

Responses to whether participants knew what CD4 results meant are presented in (c) of Table 1. Over three-quarters (513/642 (79.9%)) had a good understanding of how a CD4 result would impact their health. Almost half (44.1%) emphasized how it assessed the level of CD4 cells in their blood, with some drawing on the language of “soldier cells” to describe CD4 cells. A quarter (23.5%) emphasized how the CD4 count was used to assess whether you were eligible to start treatment or the impact of treatment on health progression. A smaller group had a more general understanding around the CD4 count being a marker of HIV, with almost one in ten (9.8%) linking it to HIV viral load, and an assessment of health status. Just 2.5% emphasized that it assessed an HIV positive status. One-fifth (20.1%) reported that they had no understanding of what a CD4 test result meant.

Participants were asked if they had a CD4 test and were eligible for ART initiation, if they felt they were ready ((d) in Table 1). Over half (374/642) (58.2%) of patients were ready to start ART if they were eligible. Almost half (44.5%) of these responses were focused on living better and being healthier. Many of these suggested a good understanding of the potential benefits of being on ART. A very small group (1.1%) reported that it was so they could be healthier to look after their family, children, or baby, recognizing the social impact of ART, while 7.3% of responses focused on the biological benefit of ART and its impact on maintaining high levels of CD4. A small group (2.3%) reported that they would feel pressured to take ART if it was necessary. Lastly, some wanted to live and not die (2.6%). Only a small minority (3.6%) were not ready to take ARVs, giving reasons that the medication would make them feel sicker, they would forget to take them, they would require counselling prior to taking ARVs, and of stigma (anxious of what others would say).

There were 13 nurses interviewed, 5 at Lancers Road and 8 at Chesterville PHC, of whom 4/5 (80%) and 6/8 (80%) were females with a median age of 31 and 45.5 years, respectively. There were 3/5 (60%) and 1/8 (12.5%%) enrolled and 1/5 (20%) and 6/8 (75%) professional nurses at Lancers Road and Chesterville PHC, respectively, while 1/5 (20%) and 1/8 (12.5%) were staff nurses at each site.

Almost half of the nurses (46.2%) ((a) in Table 2) felt that it was beneficial for patients to get their CD4 result on the same day as it would facilitate knowing their disease progression, and this was felt to help with patients’ management. Similarly, 7.7% of nurses felt it would enable patients to start ART quickly, again improving outcomes. Two nurses (15.4%) in the Chesterville PHC recognized the importance of being able to provide results on the same day for the convenience this provided to patients who may struggle to get in. One-third (30.8%) did not agree this was a good idea as the patient would have to take time off work (15.4%), and some patients were not ready for their results (15.4%). In particular, they raised the issue of being able to provide adequate counselling for patients as a potential challenge in providing results the same day.

Nurses also reflected on the impact on their workload of providing the CD4 results on the same day ((b) in Table 2). A third (30.8%) felt that it would reduce their workload as they could treat patients early and not have to see the same patients time and time again, and 15.4% of nurses also felt that it supported the patients and enabled them to start ART early by giving them clarity on their health.

In contrast, over half (53.8%) of nurses felt that it would increase the workload and not be beneficial; 30.8% described how this would increase their workload, as it would entail major administrative burdens, which would slow them down. Similarly, nearly a quarter (23.0%) were concerned that the other procedures to be completed before starting a person on ART would also lead to an increased workload.

Despite concerns about increased workload, the overwhelming majority of nurses emphasized that providing the CD4 results on the same day would help with clinical management of patients ((c) in Table 2). Almost half (46.2%) described how it would improve patient readiness, because patients would be provided with the information that they needed to understand their health. Closely associated with this would be how it may improve adherence (23.0%), with nurses being able to engage more closely with adherence counselling, as well as supporting patients in understanding the importance of adherence. One nurse (7.7%) emphasized how it would reduce follow-up visits by patients, therefore meaning that patients would not drop out. Those who were concerned about this were framed around the impact of immediate ART initiation, around requiring patients to have additional training and education (15.4%) in the context of shock of being newly diagnosed, and also requiring additional assistance from staff because of the additional tests that are needed before initiation.

The majority of nurses (92.3%) correctly interpreted a CD4 test result either as an indication of initiating treatment (46.2%), level of “soldier cells” (38.5%), or staging of HIV infection (7.7%), while 7.7% did not know the meaning of a CD4 test result ((d) in Table 2).

Nurses reported that they were able to administer ARVs. on the same day as giving a CD4 test ((e) in Table 2). Many (30.8%) reflected that this was because they had already done so for pregnant women. In addition, one (7.7%) reported that it would assist in reducing a patient’s viral load. Finally, one nurse (7.7%) also noted that the CD4 count guided the initiation of treatment. A number of nurses raised concerns about initiation on the same day, and these were related to the need to have other blood tests done before ART could be prescribed (30.8%), while one nurse (7.7%) emphasized the importance of counselling. One nurse also highlighted that ART administration was not in their job description (7.7%).

## 4. Discussion

Overall, patients and nurses were positive about the CD4 POC testing and receiving CD4 results on the same day, with both practical benefits and medical benefits being described by both groups of people. Concerns expressed about receiving CD4 results on the same day were related to worry around the “shock” of tests results, and closely related to this, the need for counselling for new patients, and further tests, before ART initiation. Understanding this more broadly, POC testing was about providing important health information and enabling improved clinical management, as well as patients being able to understand their own health and start to take charge of it.

The majority of patients were happy to receive a same-day CD4 result and would rather wait for a short period of time for results. As they would receive medical intervention immediately, there would be no loss or delay of test results, saving them time, money, and clinic accessibility, and reducing anxiety. In the current study, among those who had received CD4 test results, the length of time was from 4 days to 4 weeks, through normal laboratory systems. Two-thirds of patients had a good understanding of the meaning of a CD4 test result and were ready to start ART if eligible.

Most nurses felt that a same-day CD4 test result was beneficial to the patient, would facilitate better clinical management, and would have an impact on either reducing or increasing their workload, including initiating ART, and most gave the correct explanation of the meaning of a CD4 test result.

Regardless of POC testing exposure or not, the high acceptability by patients and clinic staff of a same-day CD4 test result confirms findings from a feasibility and acceptability study of POC EID testing [9], finger-stick blood donation [10], and POC VL testing [24]. It suggests that currently there is limited fear attached to HIV disease due to the availability of life-saving medication, and it is considered a chronic condition with little stigma attached to it. The main motivation was receiving immediate treatment due to the elimination of loss or delay of results. This study corroborates findings of POC CD4 testing in South Africa and Zimbabwe where patients appreciated receiving rapid results for quicker clinical decision-making [30,31]. Numerous studies in African cohorts [12,32,33,34,35] have described poor rates of linkage to care for those eligible for ART due to several attrition challenges in the continuum of care pathway [22,36], including the unavailability of a CD4 test result [22,37].

A small minority preferred CD4 test results on a different day, most of whom were from the Chesterville PHC, reiterating that they were not ready to receive such information immediately. As has been described [11], they wanted to internalize and come to terms with what the results of the test meant in terms of implications for their health as well as not being able to wait due to a busy clinic and work commitment. The importance of this is that POC testing needs to be combined with supportive and effective counselling to ensure that it gives people space to adjust to the news they have just received, rather than it being provided on its own.

In this study, patients expressed several practical benefits of same-day POC CD4 testing: It would save time and days off work, cause less anxiety, and be a cost saver, which have been found to be important factors resulting in loss of linkage to care if not addressed [11]. Again, these are important findings about POC, no matter what the test is for, and they highlight structural constraints within the South African healthcare system and economy that POC testing may substantially overcome.

One of the barriers was clinic inaccessibility, particularly for patients attending the Lancers Road PHC. A significantly higher percentage of patients from the Chesterville PHC clinic received routine CD4 test results within 1–7 days due to the close proximity of the clinic facility, within walking distance in the community, compared to Lancers Road of 1–4 weeks where transport was required (data not shown).

Most (80%) patients correctly explained CD4 count results related to treatment initiation due to their immune status and facilitating decrease in viral load, while 20% of patients still did not understand the meaning of CD4 count results. Our study did not provide any education; however, in South Africa, there is a long history of CD4 count testing [38], particularly through the Treatment Action Campaign, which has encouraged treatment literacy focusing on CD4 testing. With the introduction of universal coverage in South Africa and VL monitoring [2], it has been recently suggested [24] that it would be important to increase awareness through education with respect to VL results. It is therefore important to ensure that information is not just provided on health outcomes for patients, but is also provided in understandable contexts with the meaning of it for their health.

The acceptability of ART initiation if eligible was high, with patients expressing the impact of life-saving ARVs. on making them feel better in order to take care of their family, longevity, and their action on their immune system and HIV virus, while a few gave no explanation. A minority felt they had no choice and were not ready.

Both professional and enrolled nurses felt that a same-day CD4 count result was beneficial to patients as it helped determine their disease progression in order to ascertain treatment initiation and patient convenience. However, one-third felt that some patients were not ready for same-day results to start treatment or would have to take time off work due to the turnaround of results.

With respect to workload, the opinions were divided. Half of the healthcare professionals felt that it would decrease their workload as patients would be treated early whereas the remainder felt this would increase their workload as all other tests would have to be completed before ART initiation, particularly at Lancers Road PHC, the reason being that it is an extremely busy clinic with a heavy patient load [8].

With respect to clinical management, professional nurses felt that a same-day CD4 test result would facilitate patient readiness, adherence to ART, and fewer follow-up visits. We concur with findings from two qualitative studies, one from South Africa [39] and the other from Uganda [40], where healthcare workers found that POC testing resulted in earlier interventions and reduced burden of patient clinic visits. Only a minority in this study felt that they needed extra assistance and further training and education. All but one nurse (staff nurse) did not correctly interpret the meaning of a CD4 result, as this was not in her job description. Half of the nurses felt it was beneficial to have a POC CD4 count as it would facilitate ART initiation in pregnant women, as was found in a study from Zimbabwe [30]. Our findings are similar to Spooner et al. (2019) [9], in that the remainder were concerned that other tests were still required before ART initiation.

Although current programmes could be enhanced by the introduction of POC testing [41,42] to address some existing challenges [43], it has been suggested [44,45] that certain factors need to be considered before POC implementation: Clinic flows, diagnostic accuracy, staff training, quality control, and turnaround times. In South Africa, multiple strategies [46] have previously been used to overcome high patient burden and staff shortages [7] with respect to rapid HIV testing. The United Nations International Children’s Emergency Fund (UNICEF) have described the steps necessary for the incorporation of POC diagnostic testing into national health laboratory systems [47].

The strength of this study is the large patient sample size, collection of both quantitative and qualitative data from the general PHC population, its being undertaken in two different geographic settings, and the POC testing experience.

The limitations are the small sample size of healthcare professionals and the lack of inclusion of other stakeholders for their insights into the advantages and disadvantages of POC testing from their perspective. The qualitative data were from a closed-caption set of questions, and few respondents wrote more than a short sentence as an answer to any question. While in-depth qualitative interviews and focus-group discussions can generate more detailed information and understanding of a topic under consideration, the qualitative survey did allow for a large number of views to be generated from patients and some form of quantification to be done to describe the overall perceptions of patients.

## 5. Conclusions

The high patient and nurse acceptability of POC CD4 testing and their grasp of the immediate health and structural benefits (time, cost savings, and time off work) in the former and clinical management in the latter lends itself to undertaking implementation studies in the future in order to address barriers [24]. This is particularly important for POC VL testing, which is used as the hallmark for treatment success [48,49], so that the third 90 of The Joint United Nations Programme on HIV and AIDS (UNAIDS) 2020 target [50] can be achieved. It also has implications for POC testing more widely, suggesting that patients and healthcare providers recognize its wide-ranging benefit.

## Figures and Tables

**Table 1:** Patients’ understanding and perceptions of the impact of a point-of-care (POC) laboratory provision of a CD4 test result at the primary healthcare (PHC) facility on their health.

(a) Are you happy to receive a CD4 test result on the same day? (*n* = 642)	**Reasons**		**Illustrative Example**	**Overall *n* = 642 (%)**	Lancers Road PHC *n* = 322 * (%)	Chesterville PHC *n* = 320 * (%)	***p***	**Z**
	Happy	Receive medical help	I like it because I will get assistance immediately if my results say so So that I know immediately if I’m supposed to start medication	474 (73.8)	237 (73.6)	237 (74.1)	0.9083	−0.12
		Saves time/money	I live far so it cost me a lot of money to come to the clinic So I won’t have to take time off work to come to the clinic	125 (19.5)	67 (20.8)	58 (18.1)	0.3875	0.86
		No delay or loss of test results	It will avoid the loss of results and being drawn the blood again due to loss of them.	41 (6.4)	18 (5.6)	23 (7.2)	0.4075	−0.83
	Not Happy	Anxiety	I need time to get ready for the results	2 (0.3)	0	2 (0.6)	0.1645	−1.39
(b) Would you rather wait for a CD4 test result or return to clinic another day? * Not recorded for Lancers Road Clinic (*n* = 1)	Prefer to wait	Start Treatment	I would want to know if the treatment I am taking is working or not	362 (56.4)	155 (48.1)	207 (64.7)	<0.0001	−4.24
		Clinic inaccessibility	To save time and money for transport and I won’t have to come back again for results since I hate being in the clinic I will save time, money and won’t have to ask for leave at work for the second time	209 (32.6)	130 (40.4)	79 (24.7)	<0.0001	4.24
		No anxiety	If I wait for my result, I can deal with the stress same time I won’t stress about the result if I get my result same day	33 (5.1)	19 (5.9)	14 (4.4)	0.3900	0.86
		No loss of samples	I like this because sometimes I come back for nothing, they tell me my blood was lost in the lab It can prevent loss of results	15 (2.3)	9 (2.8)	6 (1.9)	0.4518	0.75
	Not Wait	Not ready for results	It’s better if I come back another day because I’m always rushing back to work Because there would be a long queue	22 (3.4)	8 (2.5)	14 (4.4)	0.1870	−1.32
(c) Do you know what a CD4 test result means?	Yes	CD4 cells/ Immune system	It tells how strong my CD4 cells I have Checking of soldier cells in the body It is your CD4 cell count.	283 (44.1)	153 (47.5)	130 (40.6)	0.0783	0.078
		Start treatment	It tells if you are ready to start treatment The results show the progress of the soldier cells in the body when taking medication.	151 (23.5)	69 (21.4)	82 (25.6)	0.2095	−1.26
		Viral load	It tells how much the virus is decreasing in the body	63 (9.8)	30 (9.3)	33 (10.3)	0.6700	−0.43
		HIV status	Tells me if I’m positive It tells me about HIV/AIDS	16 (2.5)	6 (1.9)	10 (3.1)	0.3300	−0.97
	No	Don’t know		129 (20.1)	64 (19.9)	65 (20.3)	0.8994	0.899
(d) Are you ready to start ART if eligible? *Not recorded (*n* = 3 from each PHC)	Yes	Will live/make me better	It will help me from getting other infections Because I want to be healthy and live longer So I can be able to live longer So that I can protect myself and cannot spread the virus	286 (44.5)	147 (45.7)	139 (43.4)	0.5577	0.59
		Make me better in order to look after my family	Because I want to live longer as I have a family I’m ready because I don’t want to die because I have children Because I have to, for the sake of my baby	7 (1.1)	6 (1.9)	1 (0.3)	0.0522	1.94
		Boost immune system/reduce viral load	I would start so that my CD4 levels stays high So it will increase my CD4 count	47 (7.3)	30 (9.3)	17 (5.3)	0.0515	1.95
		Ready		34 (5.3)	31 (9.6)	3 (0.9)	<0.0001	4.94
		No choice	Because I have no choice It’s a must to start treatment whether I like it or not so that I can keep a healthy lifestyle	15 (2.3)	7 (2.2)	8 (2.5)	0.8019	−0.25
		Don’t want to die	It’s my life so I have to be responsible as if I don’t take treatment, I will die I know if I don’t start treatment if my CD4 is low, I will get sick and die so I will take it	17 (2.6)	11 (3.4)	6 (1.9)	0.2370	1.18
	No	Not ready	It will be hard because I haven’t told anyone about my status because I know they won’t accept it	23 (3.6)	10 (3.1)	13 (4.1)	0.4964	−0.68
		On ARVs. already		207 (32.2)	77 (23.9)	130 (40.6)	<0.0001	−4.53

**Table 2:** Nurses’ understanding and perceptions of the impact of a POC laboratory provision of CD4 test results at the PHC facility with respect to patient workload, clinical management, and antiretroviral therapy (ART) administration/initiation.

(2a) Do you think it is beneficial for the patient to get their CD4 result on the same day?	Yes	**Reasons**	**Illustrative Example**	**Overall *n* = 13** **(%)**	**Lancers Road PHC** ***n* = 5 (%)**	**Chesterville PHC** ***n* = 8** **(%)**
		Disease progression	They get to know how their immune system is doing Because they will be informed about the progression of the disease in the body	6 (46.2)	4 (80)	2 (25)
		Convenience	Some patients work cannot afford to come to the clinic every second week. Other patients come when the clinic is very busy while they also work	2 (15.4)	0	2 (25)
		Start Treatment	Will speed up the process of initiating patients to ART and patients will get immediate care	1 (7.7)	0	1 (12.5)
	No	Time off work	If they have asked for time off at work for that day of blood draw, they might not get a chance to come another day to collect results	2 (15.4)	0	2 (25)
		Not ready for results	It’s good and bad at the same time. It’s good for someone who’s been counselled well but others may not feel so good especially if they didn’t even think of results	2 (15.4)	1 (20%)	1 (12.5)
(2b) What was the impact on your workload in giving CD4 results to the patient on the same day?	Beneficial	Reduce workload	It would make work easier and time saving because as a nurse, I won’t see the patient over and over again about something that could be done in a day. It will be easy as you know that you will be dealing with patients and finish all procedures at once rather than calling them back.	4 (30.8)	1 (20)	3 (37.5)
		Treat patients early	It gives clarity on their CD4 count on whether they are starting ARVs. or not	2 (15.4)	1 (20)	1 (12.5)
	Not beneficial	Increase workload	It will increase my work load because there’s too much administration when you initiate a person on treatment	4 (30.8)	3 (60)	1 (12.5)
		Complete all other procedures	It will increase workload; more staff like phlebotomists to take bloods. Patients will need to be prepared for ARV therapy, assess readiness and acceptance.	3 (23.0)	0	3 (37.5)
(2c) Does having a CD4 result on the same day help you with patient clinical management?	Yes	Patient readiness	Because the patients gets all the information and help he/she needs on the same day	6 (46.2)	2 (40)	4 (50)
		Adherence	It helps a lot to manage the patients who has low CD4 count	3 (23.0)	2 (40)	1 (12.5)
		Follow up visits	The patient won’t have to come to the clinic for treatment so we won’t lose the patients	1 (7.7)	0	1 (12.5)
	No	Assistance from other staff	It helps, but at the same time, it does not because there is a lot of tests that are done	1 (7.7)	0	1 (12.5)
		Training/Education	Yes, will definitely help, but on the other side, will be a disadvantage for patients who just tested positive; still a shock to them	2 (15.4)	1 (20)	1 (12.5)
(2d) Do you know what a CD4 test result mean?	Yes	Start treatment	Yes if patients have CD4 of 500 or less they are started on ART.	6 (46.2)	0	6 (75.0)
		Level of soldier cells	It tells me how high the level of soldier cells in the blood or how low the level of soldier cells in the blood	5 (38.5)	4 (80.0)	1 (12.5)
		Staging of HIV infection	It means patient is infected with the virus; gives me the picture of what stage the patient is in.	1 (7.7)	0	1 (12.5)
	No	Don’t know		1 (7.7)	1 (20.0)	0
(2e) Were you able to administer ARVs. to the patient on the same day you had the CD4 test result? _*Not recorded in Chesterville PHC for (e) (*n* = 1)_	Yes	Pregnant women	I managed the initiation of pregnant women as they are started on ARVs. immediately, regardless of their CD4 count Because I was directed by CD4 results, but with pregnant women, you provide ARVs. at the same time, regardless of CD4 count	4 (30.8)	1 (20)	3 (37.5)
		Decrease Viral load	So it would help decrease the viral load	1 (7.7)	1 (20)	0
		Treatment initiation	The CD4 count result guided me into initiating a patient	1 (7.7)	1 (20)	0
	No	Require other blood tests	A patient is not initiated immediately; he/she needs to undergo some tests first We need to check other diseases like TB, tests for liver and kidneys before initiating ARVS	4 (30.8)	2 (40)	2 (25)
		Patient education	That has not happened as yet in the clinic depending on the other things that need to be considered	1 (7.7)	0	1 (12.5)
		Not job description		1 (7.7)	0	1 (12.5)
